# Rheumatoid arthritis

**DOI:** 10.1186/s41232-020-00133-8

**Published:** 2020-09-07

**Authors:** Yoshiya Tanaka

**Affiliations:** The First Department of Internal Medicine, School of Medicine, University of Occupational and Environmental Health, Japan, Kitakyushu, 807-8555 Japan

**Keywords:** Rheumatoid arthritis, Diagnosis, Treatment, DMARD, Biological

## Abstract

Rheumatoid arthritis is an autoimmune inflammatory disease primarily characterized by synovitis which is accompanied by extra-articular organ involvement, such as interstitial pneumonia, in addition to clinical symptoms including pain, swelling, stiffness of multiple joints, fever, and malaise. Joint destruction progresses soon after the onset, and once the affected joints are deformed, the development of irreversible physical dysfunction is noted. Thus, proper diagnosis and treatment are required from the early stages of the disease. Although palliative therapy with glucocorticoids and anti-inflammatory drugs had been used, disease-modifying antirheumatic drugs (DMARDs) are currently used to suppress immune abnormalities and to control disease activity. DMARDs are classified into different groups, such as conventional synthetic DMARD, targeted synthetic DMARD, and biologic DMARD. The appropriate use of these drugs has allowed remission to be the therapeutic goal in all patients. By maintaining remission, these drugs have also been shown to prevent the progression of joint destruction and physical dysfunction over a long period. The advent of molecular-targeted therapies has allowed for the use of treatments based on pathological mechanisms, and such therapeutic strategies have also been applied to the treatment of various autoimmune inflammatory diseases. In the future, safer and more effective treatments, therapeutic strategies aimed at drug holidays or cure, and the introduction of precision medicine are expected.

## Backgrounds

Rheumatoid arthritis is an autoimmune inflammatory disease primarily characterized by synovitis. It commonly affects women in their 30s to 50s, with an incidence of 1 in 150. It is accompanied by multi-organ disorders, in addition to pain, swelling, and stiffness of multiple joints. Joint destruction progresses rapidly after onset, resulting in irreversible physical dysfunction and deformation of the affected joints. Thus, proper diagnosis and treatment are required in the early stages of the disease.

The term rheumatism comes from the 2500-year-old Greek word meaning “flowing current,” indicating the flow of the affected joints in the entire body. This disease has afflicted humanity for a long period of time, and its treatment also has a long history. There is a 2500-year-old record stating that drinking a decoction of European white willow bark alleviates pain. In the nineteenth century, salicin was discovered as a component of the bark. In 1853, Gerhardt first synthesized acetylsalicylic acid, which had superior in vivo stability to salicin, and in 1897, acetylsalicylic acid was marketed as a tablet for arthralgia by Hoffmann in Bayer and is now used other conditions worldwide. In 1949, Dr. Hench reported the first administration of cortisone to patients with rheumatoid arthritis, and its dramatic effect was widely recognized. He was awarded the Nobel Prize in Physiology or Medicine in 1950. This resulted in the use of glucocorticoids and non-steroidal anti-inflammatory drugs for the treatment of rheumatoid arthritis in the twentieth century to alleviate pain and swelling. However, disease control was inadequate, and the progression of joint destruction could not be prevented.

In the late twentieth century, rheumatoid arthritis was recognized as an autoimmune disease primarily characterized by polyarthritis. Immunosuppressive drugs were then used to correct and suppress immune abnormalities and to control disease activity. In 1984, Köhler and Milstein were awarded the Nobel Prize in Physiology or Medicine for their techniques for producing monoclonal antibodies, which were immediately applied to clinical practice. In 1998, the first monoclonal antibody therapy targeting tumor necrosis factor (TNF), a cytokine that plays an important role in rheumatoid arthritis pathogenesis, was approved. Its revolutionary clinical effect caused a paradigm shift in treatment strategies. When immunosuppressive drugs are applied for the treatment of rheumatoid arthritis, they are referred to as disease-modifying antirheumatic drugs (DMARDs). At present, DMARDs are classified into synthetic DMARDs such as methotrexate and biologic DMARDs produced from biological agents.

In the twenty-first century, the appropriate use of DMARDs allowed rheumatologists to aim for clinical remission and to control joint destruction in all patients with rheumatoid arthritis. Such therapeutic strategies are also being applied to the treatment of various autoimmune inflammatory diseases. This article provides an overview of the pathology, clinical features, diagnosis, and treatment of rheumatoid arthritis, from the basic to the latest information.

## Pathology

Genome-wide analyses of single nucleotide polymorphisms in patients with rheumatoid arthritis have identified the human leukocyte antigen D-related B1 gene (HLA-DRB1) as the most relevant disease-susceptible gene and also identified other disease-susceptible genes. These include the protein tyrosine phosphatase non-receptor type 22 (PTPN22), cytotoxic T-lymphocyte antigen-4 (CTLA4), signal transducer and activator of transcription 4 (STAT4), TNF alpha-induced protein 3 (TNFAIP3), C-C motif chemokine ligand 21 (CCL 21), and peptidyl arginine deiminase 4 (PADI4) genes. In Japanese individuals, two haplotypes of the PADI4 gene have been identified that are disease-susceptible and non-susceptible, and the messenger RNA transcribed from the disease-susceptible gene is reported to be stable. Anti-cyclic citrullinated peptide (anti-CCP) antibodies are highly disease specific, and bone or cartilage destruction is more likely to progress in patients positive for anti-CCP antibodies. In contrast, typical environmental factors, including smoking, gingivitis, and intestinal bacterial flora, can cause modulation of the epigenome and the demethylation of histones and DNA, inducing the transcription of proinflammatory cytokines. In rheumatoid arthritis, although no specific autoantigen has been identified, it is understood that the interaction between genetic and environmental factors and the citrullination of extracellular matrix molecules, such as filaggrin and fibrinogen, causes epigenetic modifications, breaking immune tolerance to antigens and inducing autoimmunity [1–3].

Autoreactive T cells and B cells accumulate in the synovial tissues of patients with rheumatoid arthritis. T cells are immunologically tolerant to autoantigens; however, when self-tolerance is broken, autoreactive T cells are activated, and they stimulate B cells to induce the production of autoantibodies. Autoantibodies form immune complexes with antigens, which are deposited in tissues and activate complements to cause histological damage (type III allergy). Tissues with synovitis are characterized by angiogenesis or vasodilation, proliferation of synoviocytes, and accumulation of lymphocytes. In tissues with diffuse inflammation, the accumulation of memory T cells and B cells can result in the formation of lymphoid follicle-like and germinal center-like structures. Here, co-stimulators and proinflammatory cytokines are highly expressed, and close cellular interactions are observed in these structures [1–3].

In synovitis lesions, lymphocytes and synoviocytes produce large amounts of inflammatory cytokines, such as TNF, interleukin (IL)-1, and IL-6, which cause synovitis. In addition to systemic symptoms, such as low-grade fever and malaise, extra-articular organ involvement, such as keratoconjunctivitis sicca, sialadenitis, and interstitial pneumonia, is often observed. Furthermore, cytokine-stimulated synoviocytes produce matrix metalloproteinases (MMP), which are released into the synovial fluid. Cartilage is degraded by these enzymes and absorbed. In addition, synoviocytes and lymphocytes express receptor activator of nuclear factor-kappa B ligand (RANKL) to induce the maturation and activation of osteoclasts. Inflammatory granulation tissues containing proliferative and stratified synoviocytes grow until they come in contact with the bones. Multinucleated osteoclasts destroy and absorb bone, causing joint destruction, mainly at the point of contact [1–5].

## Clinical features

The characteristic symptoms of rheumatoid arthritis are morning stiffness and polyarticular pain and swelling. Patients often complain of stiffness from the onset of the disease and experience difficulty in moving fingers on awakening, which is often described as having difficulty in forming a fist. Arthralgia is often associated with swelling and limited mobility. These symptoms are likely to appear in the joints of the fingers and toes (e.g., proximal interphalangeal, metacarpophalangeal, and metatarsophalangeal joints), knees, feet, hands, elbows, and cervical spine, among other areas. However, the distal interphalangeal joints are rarely the site of initial onset. In addition, patients often complain of general symptoms such as malaise, fatigue, and fever. Frequently accompanying symptoms include dry eyes associated with keratoconjunctivitis sicca (in approximately 45% of patients), xerostomia due to sialadenitis (40%), subcutaneous rheumatoid nodules on the extensor surface of the forearm (35%), numbness of the hands and feet associated with compressive neuropathy (25%), and shortness of breath on exertion or a dry cough due to interstitial pneumonia (15%).

As for the findings of clinical examinations, visual inspection and palpation tend to reveal tenderness and swelling of articular soft tissues and an accumulation of synovial fluid. Affected joints are characterized by inflammatory findings such as swelling, redness, and hot flashes. In general, multiple joints usually tend to be bilateral, symmetrical, and often mobile. As joint destruction progresses, various patterns of joint deformation are observed, such as the buttonhole deformity and swan-neck deformity of the finger joints. In case of atlantoaxial subluxation, occipital headache and numbness of the hands may occur. When inflammation spreads to the tendons, patients develop carpal tunnel syndrome due to the swelling of the trigger finger or wrist.

In terms of laboratory findings, approximately 80% of patients test positive for rheumatoid factors; however, even healthy individuals or patients with liver disease may be positive for these. Both the sensitivity and specificity of anti-CCP antibodies are 90% or higher, and patients with rheumatoid arthritis develop positivity prior to the onset of symptoms. In patients with high levels of anti-CCP antibodies or rheumatoid factors, the progression of joint destruction is rapid. Findings associated with inflammation include an elevated erythrocyte sedimentation rate and elevated C-reactive protein (CRP) levels, which are both elevated in association with disease activity. Furthermore, elevated white blood cell counts and normocytic hypochromic anemia are observed in association with inflammation. MMP-3 is a protease produced by synovial tissues and is associated with the progression of joint destruction.

Radiographic findings of the joints are important for the diagnosis and assessment of disease progression. Bone erosion localized to the affected joints is useful for diagnosis. Joint destruction is quantitatively assessed based on radiographic findings. The total Sharp score is calculated from multiple radiographs of the wrists, fingers, and toes. On these radiographs, the severity of joint space narrowing (indicating cartilage absorption) and bone erosion (indicating bone destruction) are converted into numerical scores, which are summed. Annual changes in the total Sharp scores are used to evaluate the progression of joint destruction and responses to treatment.

Meanwhile, the survival of patients with rheumatoid arthritis is considered to be shorter than that of the general population by 10 years or more owing to physical dysfunction, organ dysfunction, and adverse drug reactions. In Japanese patients, the causes of death associated with rheumatoid arthritis include respiratory dysfunction and renal failure, in addition to infection. Extra-articular organ involvement, such as interstitial pneumonia, directly affects prognosis.

## Diagnosis

**Fig. 1:**
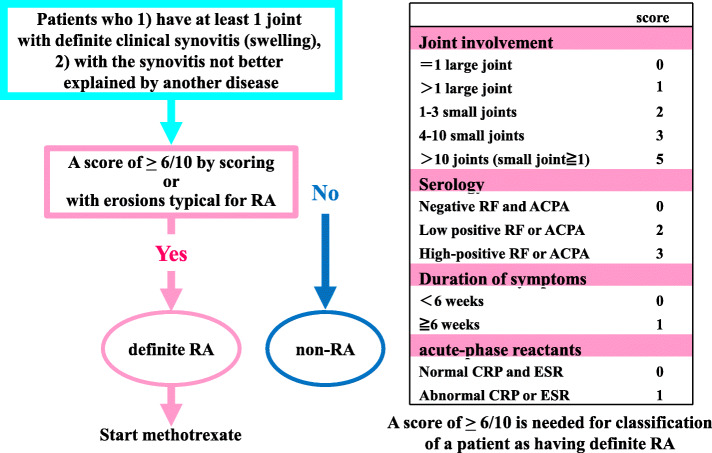
The rheumatoid arthritis classification criteria published by the ACR/EULAR in 2010. Modified from reference [6]

The rheumatoid arthritis classification criteria published by the American College of Rheumatology (ACR) and the European League Against Rheumatism (EULAR) in 2010 are widely used for diagnosis (Fig. 1) [6]. These criteria, which define rheumatoid arthritis as arthritis that is persistent and may be destructive, were formulated with the aim to differentiate it from other forms of arthritis soon after onset and to allow for prompt initiation of treatment with DMARDs. In the first step, various diseases, such as connective tissue disease accompanied by arthritis of one joint or more, osteoarthritis, spondyloarthritis, and crystal-induced arthritis, are excluded. In the second step, scores of 4 items, arthritis (swelling of the small or intermediate/large joints), serologic test results (rheumatoid factors and anti-CCP antibodies), disease duration (6 weeks or longer), and acute-phase reaction (erythrocyte sedimentation rate and CRP), are weighted and added. A condition with a score of 6 points or higher out of 10 points is classified as definite rheumatoid arthritis. In addition, arthritis affecting one joint or more which is accompanied by typical bone erosion is also classified as rheumatoid arthritis, regardless of the score. When rheumatoid arthritis is comprehensively diagnosed based on the classification criteria, treatment with DMARDs is initiated. This diagnostic process potentially allows for therapeutic intervention prior to joint destruction.

The assessment of disease activity is essential for planning therapeutic strategies. The 28-joint Disease Activity Score (DAS28), which is calculated based on the number of tender or swollen joints among 28 specified joints, 1-h erythrocyte sedimentation rate, and the patient’s global assessment of disease activity with a dedicated formula, is widely used for the objective assessment of disease activity. DAS28 scores are interpreted as follows: > 5.1, high disease activity; 3.2–5.1, moderate disease activity; < 3.2, low disease activity; and < 2.6, remission. Likewise, the Simplified Disease Activity Index (SDAI) and the Clinical Disease Activity Index (CDAI) are also widely used. For the evaluation of physical dysfunction, the Health Assessment of Questionnaire Disability Index, which consists of 20 questions on 8 categories regarding physical function in daily living, is widely used worldwide.

As described above, rheumatoid arthritis is often complicated by extra-articular involvement of the eyes, oral cavity, blood, lungs, heart, skin, nerves, kidneys, and lymph nodes, among others. Lung disorders are important organ dysfunctions that affect prognosis. Chest computed tomography (CT) is used to reveal lung disorders in approximately 70% of patients. Of these patients, approximately 50% are considered to present with nonspecific changes, approximately 30% are considered to present with interstitial pneumonia, and approximately 20% are considered to present with chronic infection or chronic obstructive pulmonary disease. Other pathological conditions that can occur include pleurisy, pulmonary alveolar hemorrhage, and bronchiectasis [1–3]. In cases of rheumatoid vasculitis, in which progressive arthritis is accompanied by systemic vasculitis in the skin, gastrointestinal tract, heart, lungs, spleen, and pleura, in addition to interstitial pneumonia. Furthermore, as the disease activity of rheumatoid arthritis increases, lymphoproliferative disease may concomitantly occur. Patients may also concomitantly develop various autoimmune diseases, including Hashimoto’s disease, other thyroid diseases, and secondary Sjögren’s syndrome. In all cases, conditions such as organ dysfunction associated with the pathology of rheumatoid arthritis, other comorbidities, concomitant infections with bacteria and viruses, and adverse events caused by drugs should be differentiated.

## Treatment

The basic policy of the treatment of rheumatoid arthritis involves immediate intervention after diagnosis, before the onset of joint destruction, to suppress arthritis and induce remission. Therapeutic strategies should be determined based on a comprehensive assessment of disease activity, imaging findings (such as radiography findings), complications, and comorbidities. Composite objective indices, such as the SDAI, CDAI, and DAS28, are widely used to evaluate disease activity. The therapeutic goal is remission, defined as a clinical condition involving no progression of either joint destruction or dysfunction in the future. Boolean remission and numerical targets, such as an SDAI score ≤ 3.3 and a CDAI score ≤ 2.8, have been statistically established as remission criteria [7].

**Fig. 2:**
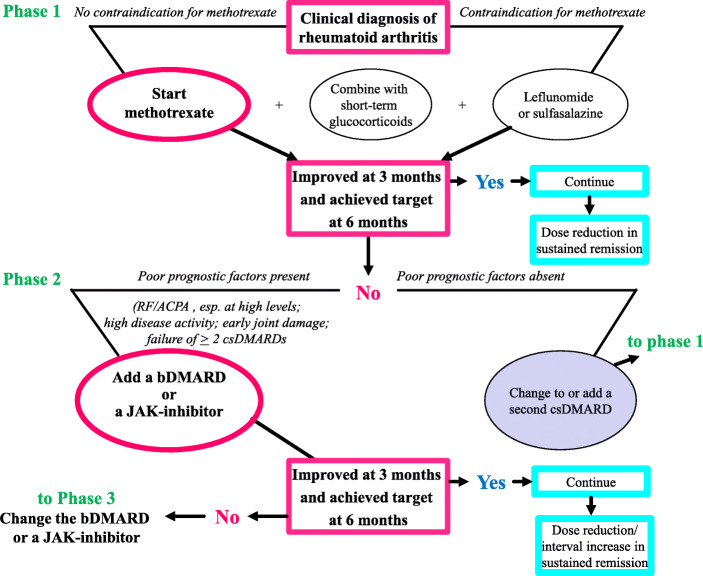
EULAR recommendations for the management of rheumatoid arthritis with synthetic and biological DMARDs: 2019 update. Modified from reference [8]

In the standard initial treatment after the diagnosis of rheumatoid arthritis, methotrexate, a conventional synthetic DMARD, should be used if it is not contraindicated [8, 9]. However, when no improvement is observed within 3 months or when no remission is achieved within 6 months, despite an increase to the full dose of methotrexate, the addition of biological DMARDs or Janus kinase (JAK) inhibitors is recommended. If the therapeutic goal is still not achieved, biological DMARDs or JAK inhibitors should be altered approximately 3–6 months later. Meanwhile, glucocorticoids are recommended for temporary use for up to 3 months as adjunctive therapy to relieve pain and swelling at the time of initial onset or the relapse of arthritis (Fig. 2).

While more than 10 conventional synthetic DMARDs have been approved, methotrexate is recommended as the first choice of standard drug therapy to be administered after the diagnosis of rheumatoid arthritis, if its use is not contraindicated. Methotrexate exerts antirheumatic effects mainly by controlling the proliferation of lymphocytes and synoviocytes in the mitotic phase via antagonistic action against folic acid. It is more effective than any other conventional synthetic DMARD. Adverse reactions to methotrexate include liver dysfunction and gastrointestinal dysfunction. In elderly patients, attention should be paid to myelosuppression, interstitial pneumonia, opportunistic infection, and lymphoproliferative disease. Co-administration of folic acid is useful for reducing adverse reactions. Sulfasalazine and leflunomide are recommended when methotrexate use is contraindicated.

Biological DMARDs are selected when responses to synthetic DMARDs are inadequate. In Japan, TNF-targeting drugs (i.e., infliximab, etanercept, adalimumab, golimumab, and certolizumab), IL-6-targeting drugs (i.e., tocilizumab and sarilumab), and the T cell-selective co-stimulation modulator abatacept can be administered by injection or drip infusion. All of these drugs exert prompt and potent clinical effects. Their use in combination with methotrexate allows the induction of remission in approximately half of the cases. Biological DMARDs can also prevent the progression of joint destruction and dysfunction for long periods of time [10].

In contrast, inhibitors against JAKs, which are intracellular signaling molecules such as cytokines, are classified as targeted synthetic DMARDs. Tofacitinib, baricitinib, peficitinib, upadacitinib, and filgotinib are used for the treatment of rheumatoid arthritis and differ in their selectivity for different JAK isoforms [11–15]. Although they are all orally administered drugs, they have multi-target effects and exert clinical effects just as promptly as biological DMARDs. JAK inhibitors can be used alone or in combination with methotrexate.

In Japan, when biological DMARDs were used for the treatment of rheumatoid arthritis, post-marketing surveillance was required to verify their safety [16, 17]. According to an all-case surveillance study on infliximab administered to 5000 patients over a period of 6 months, adverse reactions occurred in 1401 patients, and serious adverse reactions occurred in 308 patients, including bacterial pneumonia in 108 patients, interstitial pneumonia in 25, pneumocystis pneumonia in 22, and tuberculosis in 14. The risk factors for pneumonia due to the use of biological DMARDs include advanced age, a history of respiratory diseases, and concomitant use of glucocorticoids. Owing to these factors, the use of biological DMARDs requires medical management and treatment for serious adverse reactions such as pneumonia, tuberculosis, and other opportunistic infections, and guidelines for the prevention and treatment of adverse reaction have been established. For example, the prophylactic administration of isoniazid is recommended for patients with risk factors for tuberculosis, and pneumococcal vaccination is recommended for patients with risk factors for pneumonia.

In addition, JAK inhibitors should not be used without careful consideration as they are orally administered drugs with multi-target effects based on the inhibition of intracellular signaling. Screening before their use and monitoring during treatment should be strictly performed. They should be administered by physicians who can perform systemic management in the event of adverse events. JAK inhibitors should not be used in patients with serious infections, liver disorders, renal disorders, or blood cell disorders, and it is necessary to establish evidence on its long-term safety regarding the development of infections such as herpes zoster and malignant tumors such as lymphoma.

In our department, approximately 4000 patients have been treated with biological DMARDs that were introduced into or substituted for other drugs in the FIRST registry since 2003. According to the clinical pathway, these patients were admitted and examined for contraindications and factors requiring caution; then, they were carefully evaluated as to whether they showed indications for the use of these drugs. In addition, the safety and efficacy of the drugs in these patients were rigorously monitored through outpatient visits for over 1 year. In particular, CT from the head to the abdomen detected early lung cancer in 11 patients and non-tuberculous mycobacteriosis in 13 patients from among approximately 2500 patients before they became symptomatic. This demonstrates the importance of in-depth screening.

## Development

New therapeutic systems and strategies for rheumatoid arthritis have been applied to other connective tissue diseases and rheumatic diseases. While their indications have been expanded, these systems and strategies have also led to breakthroughs in treatment in each field. Infliximab, a TNF-targeting drug, was initially indicated for rheumatoid arthritis, but this indication has been expanded to the treatment of more than 10 immune diseases, such as Behcet’s disease, Kawasaki disease, psoriasis, psoriatic arthritis, ankylosing spondylitis, Crohn’s disease, and ulcerative colitis. Similar trends have been observed for other TNF-targeting drugs such as adalimumab. Treatment with TNF-targeting drugs prevented the loss of vision due to uveitis in the majority of patients with Behcet’s disease and dramatically reduced the development of fistula formation in Crohn’s disease patients with inflammatory bowel disease. Furthermore, tocilizumab, an IL-6-targeting drug, was found to exert marked effects on juvenile idiopathic arthritis. Its indication has been expanded to include Castleman’s disease and cytokine-release syndrome associated with chimeric antigen receptor T cell therapy, in addition to adult-onset Still’s disease, Takayasu’s arteritis, and giant cell arteritis. Tocilizumab has also been determined to be very effective for all of these conditions.

As various molecular-targeted drugs are used for many autoimmune diseases, it is necessary to develop new therapeutic strategies involving the differential use of drugs. This is especially important for highly diverse autoimmune diseases. Although biological drugs targeting TNF, IL-17, and IL-12/IL-23 have been approved for the treatment of psoriatic arthritis with destructive spondyloarthritis, there is no way to differentiate the use of these drugs. In our department, 8-color flow cytometry is performed to analyze the phenotypes of peripheral blood lymphocytes of patients with psoriatic arthritis registered in the FLOW registry [18, 19]. The patients were classified into four groups based on the expression of chemokine receptors: helper T cell (Th) 17-dominant, Th1-dominant, hybrid, and normal type. Patients with Th17-dominant type were treated with an IL-17 antibody, patients with Th1-dominant type were treated with a p40 antibody, and patients with hybrid or normal type were treated with TNF-targeting drugs. The proportion of patients without improvement was reduced to less than 10% in these patients, compared with that in patients who were conventionally treated with biological drugs. Thus, differential use of biological drugs was demonstrated to be highly effective. This result suggested that for diseases in which characteristic cytokines are involved in the pathology, the use of molecular-targeted drugs can be optimized according to the pathology by stratification based on lymphocyte analysis. In other words, the applicability of precision medicine was suggested. These findings are expected to contribute to the development of new therapeutic systems and strategies.

For the treatment of rheumatoid arthritis, safe and favorable maintenance therapy is required over a long period after remission induction with methotrexate and biological DMARDs. However, the burden of medical expenses and medical economic problems due to the long-term continuous use of drugs are urgent issues in Japan and overseas, and the safety of long-term inhibition of targets, such as TNF, is currently unknown. Dose reduction and extension of dosing intervals for biological DMARDs are associated with a lower incidence of relapse than drug withdrawal, but there is a concern that anti-drug antibodies are more likely to be produced in such situations. If biological DMARDs can be withdrawn, adverse events should be preventable. The RRR study and the HONOR study reported the possibility of withdrawal of biological DMARDs after the induction of remission in patients with rheumatoid arthritis [20, 21].

**Fig. 3:**
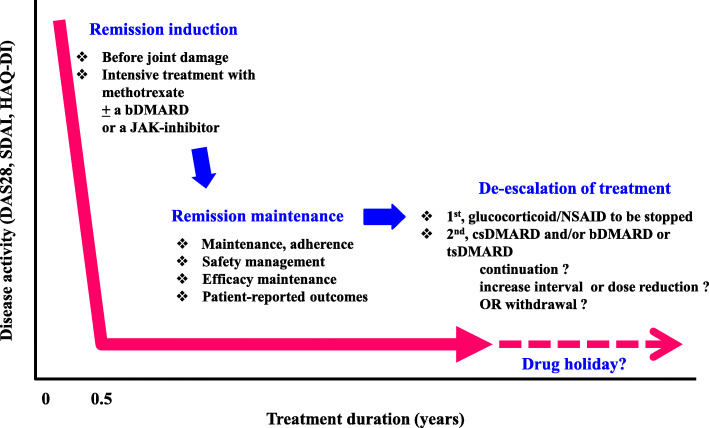
Strategies for the treatment of rheumatoid arthritis. Intensive treatment is required for inducing remission in rheumatoid arthritis, but subsequently maintaining remission with high adherence and safety is a prerequisite for the good long-term outcome. The de-escalation and drug holiday of the DMARDs is an extension of the maintained remission

At the 2016 International Round-table Conference, study results from Japan and overseas were reviewed, and a consensus was reached regarding the order of drug withdrawal. The drugs should be withdrawn in the following order: glucocorticoids, anti-inflammatory drugs, biological DMARDs, and finally synthetic DMARDs. In addition, four requirements were determined for the withdrawal of DMARDs: fulfillment of the standard remission criteria, maintenance of remission for at least 6 months, maintenance of treatment with the same drugs at the same doses for at least 6 months, and no use of glucocorticoids. Moreover, it was additionally stated that negativity for anti-CCP antibodies, deep remission, and the absence of ultrasound findings of synovitis are all associated with the possibility of remission after withdrawal of DMARDs [22]. This suggests that if remission can be maintained even after the withdrawal of biological DMARDs, drug-free remission can be subsequently achieved (Fig. 3) [23, 24]. It is also suggested that if the pathological process is controlled, a cure can be achieved by resetting immune abnormalities while the causes remain in place. The establishment of a new therapeutic system involving a drug holiday is expected to contribute to a reduction in treatment costs and a resolution of medical economic problems.

## Conclusions

Rheumatoid arthritis is an autoimmune inflammatory disease pathologically characterized primarily by synovitis. Joint destruction, which is associated with prolonged arthritis, progresses soon after the onset of disease. The deformation of affected joints is irreversible and causes physical dysfunction. Thus, proper diagnosis and treatment are required from the early stages. The classification criteria published by the ACR and EULAR in 2010, which define rheumatoid arthritis as arthritis that is persistent and can be destructive in the future, were formulated with the aim of differentiating it from other types of arthritis soon after onset and to allow for therapeutic interventions prior to joint destruction. For treatment, DMARDs are used to suppress immune abnormalities and control disease activity. DMARDs are classified into conventional synthetic DMARDs (e.g., methotrexate), targeted synthetic DMARDs (e.g., JAK inhibitors), and biologic DMARDs. Appropriate treatment with these drugs has allowed clinicians to aim for remission in rheumatoid arthritis patients. These drug classes have been demonstrated to prevent structural damage to the joints and to prevent the progression of physical dysfunction. The advent of molecular-targeted drugs, such as biological drugs and JAK inhibitors, has allowed for the use of targeted therapies based on pathological mechanisms and the management of autoimmune inflammatory diseases, which were previously considered to be intractable. This can be regarded as revolutionary progress. In the future, safer and more effective treatments, therapeutic strategies aiming at cure, and the introduction of precision medicine are expected. Translation research aimed at developing new therapies and preventative measures may provide motivation for young clinicians and researchers.

## Data Availability

Not applicable.

## Abbreviations

ACR: American College of Rheumatology
CDAI: Clinical Disease Activity Index
CRP: C-reactive protein
CT: Computed tomography
DMARDs: Disease-modifying antirheumatic drugs
EULAR: European League Against Rheumatism
JAK: Janus kinase
MMP: Matrix metalloproteinase
SDAI: Simplified Disease Activity Index
TNF: Tumor necrosis factor
